# Does learning style preferences influence academic performance among dental students in Isfahan, Iran?

**DOI:** 10.3352/jeehp.2018.15.8

**Published:** 2018-03-24

**Authors:** Najmeh Akhlaghi, Hosein Mirkazemi, Mehdi Jafarzade, Narjes Akhlaghi

**Affiliations:** 1 Dental Research Center, Department of Pediatric Dentistry, School of Dentistry, Isfahan University of Medical Sciences, Isfahan, Iran; 2 School of Medicine, Tehran University of Medical Science, Tehran, Iran; Hallym University, Korea

**Keywords:** Academic performance, Dental students, Iran, Learning methods, Problem-based learning

## Abstract

**Purpose:**

The present study aimed to identify the learning preferences of dental students and to characterize their relationship with academic performance at a dental school in Isfahan, Iran.

**Methods:**

This cross-sectional descriptive study included 200 undergraduate dental students from October to November 2016. Data were collected using a 2-part questionnaire. The first part included demographic data, and the second part was a Persian-language version of the visual, aural, read/write, and kinesthetic questionnaire. Data analysis was conducted with the chi-square test, 1-way analysis of variance, and multiple linear regression.

**Results:**

The response rate was 86.6%. Approximately half of the students (51.5%) had multimodal learning preferences. Among the unimodal group (48.5%), the most common mode was aural (24.0%), followed by kinesthetic (15.5%), reading-writing (8.0%), and visual (1.0%). There was a significant association between academic performance and the reading/writing learning style preference (P< 0.01).

**Conclusion:**

Multimodal learning styles were the most preferred. Among single-mode learning styles, the aural style was most common, followed by the kinesthetic style. Students with a reading/writing preference had better academic performance. The results of this study provide useful information for preparing a more problem-based curriculum with active learning strategies.

## Introduction

Learning style has been described as a complicated process in which the learner should perceive, process, save, and recall concepts efficiently and effectively [1]. Learning style has been considered to be a combination of cognitive, emotional, and physiological factors that are influenced by environmental factors [2]. Several methods have been developed to measure learning styles, including the validated visual, aural, read/write, and kinesthetic (VARK) questionnaire created by Fleming [2]. He defined the 4 sensory aspects as follows. Visual learners learn by seeing figures, diagrams, films, and maps. Aural learners learn by listening to lectures, discussions, and speeches. Learners with a reading/writing style learn by reading books, course notes, and notes. Kinesthetic learners learn by touching and experience, physical actions, and working with their hands.

Students’ learning preferences may be influenced by several factors, including gender, age, major, and sociocultural factors [3]. Contradictory results regarding these potential influences have been reported in various studies [3-6]. If students become aware of their learning style preference, they can learn more information in a shorter time [7]. Therefore, it is important to identify students’ learning style preferences in order to design an effective educational curriculum. The present study aimed to identify learning preferences among dental students in Isfahan, Iran and to characterize the associations between learning preferences and past academic performance, as represented by students’ grade point average (GPA).

## Methods

### Ethical statement

This study was approved by the research committee of the dentistry department of Isfahan University of Medical Sciences (approval #394850). After explaining the purpose of the study to students, informed written consent was obtained. Participation in this study was optional, and personal information and all responses were kept confidential. Respondents were assured of their right to withdraw from the study at any time. The GPA was taken by a staff member of the college educational administration based on the student ID number in separate forms. Then the results were analyzed by the researcher (NAR A) who did not know the students or their ID numbers.

### Study design

This research was performed as a descriptive-cross sectional study from October to November 2016 at the Dental School of Isfahan University of Medical Sciences, Isfahan, Iran.

### Materials and/or subjects

The participants consisted of undergraduate dental students, and were divided into 2 groups, depending on whether they were in the preclinical course (second-year) or the clinical course (fourth, fifth and sixth year). First-year dental students were not included because they had not yet attained a GPA. Students who chose not to complete the questionnaire were excluded as well.

### Technical information

To maximize the representativeness of the data, all the second-, fourth-, fifth-, and sixth-year dental students (n= 231) were selected using the census method. A total of 200 students answered the questionnaire from the target sample of 231.

After providing informed written consent, the participants were invited to respond to the questionnaires anonymously. The participants were visited during their regular classes by the researchers, who handed them the questionnaires, and then the questionnaires were collected once complete. Before filling out the questionnaire, the necessary information was explained by the researchers.

### Study instrument

Data were collected using a 2-part questionnaire. The first part included questions on demographic data, such as age, gender, marital status, stage of the degree program (preclinical or clinical), and academic performance data. Academic achievement was measured by GPA, which accessed from the students’ educational records based on their student identification number. The second part was the VARK questionnaire developed by Fleming [2], which was used to determine students’ learning preferences. The latest English version of the VARK questionnaire was used in this study (VARK 7.1). The VARK questionnaire consists of 16 questions with 4 options each.

In this study, the instrument was administered using a printed form that was downloaded from the official VARK website. All choices corresponded to the 4 sensory modalities measured by the VARK questionnaire (visual, aural, reading/writing, and kinesthetic). The participants were asked to select the choice that best described their priority for each item; they were permitted to mark more than 1 choice, if necessary. In addition, they were asked not to answer questions that they did not understand. The English questionnaire has been used with students in many studies around the world [2,7-9] and its validity and reliability have been assessed.

#### Validity and reliability of the Persian questionnaire

The English version of the VARK questionnaire was translated into Persian, the students’ native language. In Persian, words may have different meanings. Therefore, the translated VARK questionnaire was first tested for reading comprehension in a group of students who were not part of the group analyzed in the present study. The translated questionnaire maintained the original lettering order of the 4 choices in each question.

To verify the face and content validity of the questionnaire, it was submitted to 5 expert members of the pediatric dentistry faculty. Then, the answers were computed according to the following content validity ratio (CVR) formula: CVR= , $\frac{nE-N/2}{N/2}$, where nE is the number of experts who selected the question as necessary, and N is the total number of experts.

After the answers of the experts were collected, questions with a CVR score lower than 0.42 were excluded [10]. The number of questions in the primary questionnaire was 16. One question (“You are not sure whether a word should be spelled ‘dependent’ or ‘dependant’. You would:”) was excluded after CVR review, because in the experts’ opinion, the Persian translation of this question was meaningless without using the English word. As we used the forward-backward translation method, the Persian translation was returned to English again and reviewed by a language expert. Therefore it was not applicable to use a word with different spells in Persian (for example use letters such as ذ ,ض ,and ظ in a Persian word) in translatation from Persian into English.

Fifteen questions were finally approved by the experts, who were then asked about face validity. The questionnaire was pilot-tested on 20 students to evaluate its comprehensibility. The Cronbach alpha was used to ensure the reliability of the attitude-related questions after pilot-testing, and reliability was confirmed (Cronbach alpha=0.86).

#### Data analysis

The VARK questionnaire was analyzed using the stepping-stone method explained on the VARK website [2]. The students were classi- fied according to their preferred sensory modality for learning as visual, aural, reading/writing, or kinesthetic learners. The preferred sensory modality was the modality that received the highest score in each individual VARK questionnaire [2]. The number of students who preferred each mode of learning was divided by the total number of responses to determine the percentage of students in each category.

### Statistical analysis

Data were analyzed using IBM SPSS ver. 21.0 (IBM Corp., Armonk, NY, USA). The data were reported as the percentage of respondents in each learning preference category. Analysis of variance (ANOVA) was used to investigate differences in GPA and students’ learning preferences. The chi-square test was performed to compare the distributions of learning preferences by gender and marital status. GPA was classified into 3 groups, and the chi-square test was performed to investigate the relationship between GPA and students’ learning preferences. The Fisher exact test was used when the chi-square test was not suitable. P-values < 0.05 were considered to indicate statistical significance. Multiple linear regression was used to examine the relationship between GPA as a dependent variable and learning preferences, after adjusting for variables used in other studies.

## Results

Of the 231 undergraduate dental students invited to participate in the study, 200 students answered the questionnaire. The response rate was 86.6%. Table 1 presents a comparison of learning preferences and characteristics among dental students. Raw data are available from Supplement 1. Half of the students (51.5%) had a multimodal learning preference. The VARK mode distribution among students was unimodal, followed by bimodal, quad-modal, and trimodal modal with the percents of 48.5, 22.5, 15.5, and 13.5, respectively.

**Fig. 1.**
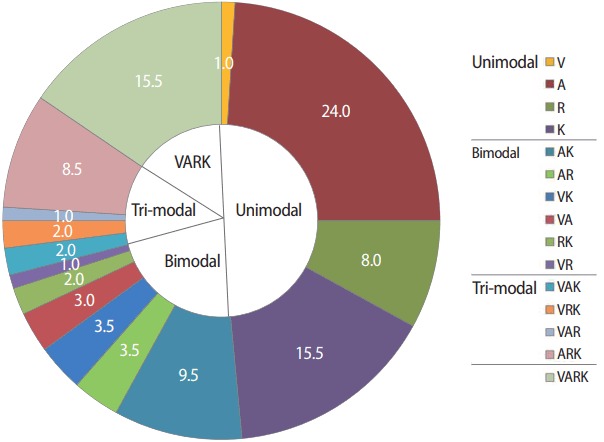
Percentages of unimodal learners who preferred a single mode of learning (V, A, R, and K), and learners who preferred 2 (bimodal) and 3 (tri-modal) (n = 200). V, visual; A, aural; R, reading/writing; K, kinesthetic; VARK, visual, aural, read/write, and kinesthetic questionnaire.

The distribution of learning preferences is shown in Fig. 1.

Table 2 shows the distribution of students’ preferred learning styles and mean GPA, as analyzed using ANOVA. No statistically significant relationship was found between GPA classification and learning style preferences based on the Fisher exact test (P=0.15) (Table 1). There was no significant difference in individual sensory modality preference among unimodal learners based on ANOVA (P= 0.07). However, there was a significant association between GPA and the reading/writing learning style preference (P= 0.01) (Table 3). Learning style preferences did not differ between male and female students according to the chi-square test (P= 0.95). No significant relationship was found between marital status and learning style preference (P= 0.43, F-test=2.4)

There was no significant difference in learning style preferences according to whether students were in the preclinical or clinical course (P= 0.79, F-test= 2.4). As secondary results, using multiple linear regression analysis, the following findings were obtained. A significant relationship was found between gender and GPA, with females having a higher GPA (P= 0.005). Younger students had a significantly higher GPA (P=0.001). There was a significant relationship between marital status and GPA, with married students having a higher GPA (P= 0.025).

## Discussion

In the present study, half of the students had multimodal learning preferences (2–4 styles), and 48.5% preferred a single-mode learning style. The most common learning preferences in this study were unimodal, followed by bimodal, quad-modal, and tri-modal. In this study, students had different learning preferences, which mean that educators must use multi-sensory engaged learning strategies to be inclusive of all types of learning preferences and modes of metacognition. The aim of this study was to determine the relationship between learning style preference and GPA using the standard VARK questionnaire, and to assess whether this relationship was affected by gender, marital status, and students’ year of study in the curriculum.

Since the students reported several learning style preferences, it was difficult to investigate the relationship between every student’s learning style(s) and her/his GPA. Therefore, we investigated the relationship between students’ GPA and having or not having one of the visual, aural, reading/writing, and kinesthetic learning styles. One of the main findings of this study was that individuals with a reading/ writing learning style preference had better academic performance than individuals without a reading/writing learning style preference. However, no statistically significant difference was found in GPA between students with different learning style preferences considered separately. This lack of significance might be due to the fact that GPAs were similar in students with different learning preferences. At the Isfahan Dental School, the teaching method for theoretical courses features passive lectures with PowerPoint (aural, visual) in large classes. Students make notes in classes (reading/writing style). However, in the present study, only 1%, 24%, and 8% of the students were found to be visual, aural, and kinesthetic learners, respectively. Therefore, it seems that the compatibility between teaching methods and students’ learning styles at this college must be reviewed. This observation encouraged us to propose reductions in passive lecture hours and to prepare a more problem-based curriculum.

According to previous research [11], the use of active learning strategies lead to better student learning, because such strategies involve a variety of learning styles, enhance learners’ thinking and reasoning, and improve skills such as problem-solving and decision-making. Discussions, small-group learning exercises, role-playing, simulations, models, challenges, and games are active teaching methods that can be utilized in a class with large number of students. A limited number of studies have evaluated the relationship between learning style preferences and academic performance among dental students using the VARK method. In 4 studies [1,8,9,11,12], no significant relationship was found between learning style preferences and academic achievement. However, Al-Saud [6] reported that there was a significant relationship between GPA and learning style preference. A higher mean GPA was found among first-year dental students with the quad-modal learning style preference.

This study found no significant relationship between learning preference and gender, which is consistent with the results of the study by Bennadi et al. [13]. However, Al-Saud [6] and AlQahtani et al. [14] found that males and females preferred different styles in Saudi Arabia. In the study by Sarabi-Asiabar et al. [3], males tended to have the kinesthetic style and females tended to learn using the aural style. Nonetheless, based on these contradictory results, it seems that if there are real differences in learning preferences related to gender, the differences are minor.

Based on the findings of the present study, it can be concluded that students’ learning preferences are not influenced by factors such as age, gender, and marital status. In the clinical departments of our dental school, students are trained by touch and experience, physical action, and working with their hands (kinesthetic style). However, no significant difference was found between preclinical and clinical students regarding their learning preference; this result is consistent with those of the research by Asiry [15] on dental students.

Among the limitations of the present study, it can be mentioned that the study was conducted in only a single dental school and may therefore not be generalizable to schools with students of different ethnicities/cultures and schools that use different academic methods.

This study was cross-sectional, and it is therefore recommended to conduct longitudinal research to investigate the possibility that learning preferences may change over time. In conclusion, the preferred learning style of undergraduate dental students in our institution was multimodal. The aural style had the highest percentage among the single-mode learning styles, followed by the kinesthetic style. There was a significant relationship between the reading/writing learning style preference and academic performance. The results of this study provide useful information for proposing reductions in passive lecture hours and preparing a more problem-based curriculum using active learning strategies. The learning preferences of the students were not influenced by age, gender, or marital status.

## Figures and Tables

**Table 1.** Comparison of learning styles among undergraduate dental students based on demographic information (n=200)

Variable	Unimodal				Bimodal	Tri-modal	VARK	Sum	P-value
	V	A	R	K					
No. of students	2 (1)	48 (24)	16 (8)	31 (15.5)	45 (22.5)	27 (13.5)	31 (15.5)	200 (100)	
Gender^a)^									0.95^b)^
Male	1 (1.1)	19 (21.8)	8 (9.2)	15 (17.2)	21 (24.1)	10 (11.5)	13 (14.9)	87 (43.5)	
Female	1 (0.9)	29 (25.7)	8 (7.1)	16 (14.2)	24 (21.2)	17 (15)	18 (15.9)	113 (56.5)	
Marital status^a)^									0. 43^b)^
Single	2 (1.2)	40 (23.8)	12 (7.1)	27 (16.1)	36 (21.4)	26 (15.5)	25 (14.9)	168 (84)	
Married	0	8 (25)	4 (12.5)	4 (12.5)	9 (28.1)	1 (3.1)	6 (18.8)	32 (16)	
Students’grade level^a)^									0.79^b)^
Pre-clinical (year 2)	0	15 (31.3)	4 (8.3)	6 (12.5)	11 (22.9)	7 (14.6)	5 (10.4)	48 (24)	
Clinical (years 4–6)	2 (1.3)	33 (21.7)	12 (7.9)	25 (16.4)	34 (22.4)	20 (13.2)	26 (17.1)	152 (76)	
Grade point average (mean)^a)^									0.15^b)^
12–14.99	1 (3.1)	6 (18.8)	1 (3.1)	4 (12.5)	12 (37.5)	2 (6.3)	6 (18.8)	32 (16)	
15–16.99	1 (0.9)	32 (27.6)	9 (7.8)	15 (12.9)	23 (19.8)	15 (12.9)	21 (18.1)	116 (58)	
17–20	0	10 (19.2)	6 (11.5)	12 (23.1)	10 (19.2)	10 (19.2)	4 (7.7)	52 (26)	

Values are presented as number (%).

a) By Fisher test.

b) No statistically significant difference was found.

V, visual; A, aural; R, reading/writing; K, kinesthetic; VARK, visual, aural, read/write, and kinesthetic questionnaire.

**Table 2.** Frequency distribution of students’ preferred learning styles and mean GPA (analysis of variance)

Learning preference	Number	GPA			
		Mean ± standard deviation	95% confidence interval for mean	Min	Max
Visual	2	14 ± 2.82	11.41–39.41	12	16
Aural	48	15.92 ± 1.2	15.57–16.27	12.25	19
Reading/writing	16	16.53 ± 1.29	15.84–17.22	14.5	19
Kinesthetic	31	15.99 ± 1.85	15.31–16.67	12	18.5
Bimodal	45	15.76 ± 1.22	15.4–16.13	13.7	18.89
Trimodal	27	16.06 ± 1.32	15.53–16.58	12	18
VARK	31	15.49 ± 1.01	15.12–15.87	13	17.43
Total	200	15.88	15.69–16.07	12	19

GPA, grade point average; VARK, visual, aural, read/write, and kinesthetic questionnaire.

**Table 3.** Relationships between learning style preference (unimodal) and GPA among dental students

Student’s learning preference	Frequency (no.)	GPA (mean±standard deviation)	P-value
Visual			
Visual style	2^a)^	14.00 ± 2.82	
Not visual style	198^a)^	15.95 ± 1.17	
Aural			0.74^b)^
Aural style	48	15.92 ± 1.20	
Not aural style	152	15.63 ± 1.58	
Reading/writing			0.01c)
Reading/writing style	16	16.53 ± 1.29	
Not reading/writing style	183	15.53 ± 1.57	
Kinesthetic			0.21^b)^
Kinesthetic style	31	15.99 ± 1.85	
Not kinesthetic style	169	15.62 ± 1.47	

GPA, grade point average.

a) Comparing 2 versus 198 was not appropriate for visual and non-visual learners.

b) No statistically significant difference was found. c)A statistically significant difference was found.

## Supplementary materials

Supplement 1. Data files are available from https://doi.org/10.7910/DVN/WFBF0U

Supplement 2. Audio recording of the abstract.

## References

[1] Jiraporncharoen W, Angkurawaranon C, Chockjamsai M, Deesomchok A, Euathrongchit J (2015). Learning styles and academic achievement among undergraduate medical students in Thailand. J Educ Eval Health Prof.

[2] Fleming N (c2001-2013). VARK a guide to learning styles: frequently asked questions [Internet]. http://www.vark-learn.com/english/page.asp.

[3] Sarabi-Asiabar A, Jafari M, Sadeghifar J, Tofighi S, Zaboli R, Peyman H, Salimi M, Shams L (2014). The relationship between learning style preferences and gender, educational major and status in first year medical students: a survey study from iran. Iran Red Crescent Med J.

[4] Peyman H, Sadeghifar J, Khajavikhan J, Yasemi M, Rasool M, Yaghoubi YM, Nahal MM, Karim H (2014). Using VARK approach for assessing preferred learning styles of first year medical sciences students: a survey from Iran. J Clin Diagn Res.

[5] Omar E (2017). Perceptions of teaching methods for preclinical oral surgery: a comparison with learning styles. Open Dent J.

[6] Al-Saud LM (2013). Learning style preferences of first-year dental students at King Saud University in Riyadh, Saudi Arabia: influence of gender and GPA. J Dent Educ.

[7] Shenoy N, Shenoy K A, U P R (2013). The perceptual preferences in learning among dental students in clinical subjects. J Clin Diagn Res.

[8] Almigbal TH (2015). Relationship between the learning style preferences of medical students and academic achievement. Saudi Med J.

[9] Liew SC, Sidhu J, Barua A (2015). The relationship between learning preferences (styles and approaches) and learning outcomes among pre-clinical undergraduate medical students. BMC Med Educ.

[10] Saber A, Tabatabaei SM, Akasheh G, Sehat M, Zanjani Z, Larijani B (2017). Face and content validity of the MacArthur competence assessment tool for the treatment of Iranian patients. Int J Prev Med.

[11] Gadbury-Amyot CC, Redford GJ, Bohaty BS (2017). Dental students’ study habits in flipped/blended classrooms and their association with active learning practices. J Dent Educ.

[12] Urval RP, Kamath A, Ullal S, Shenoy AK, Shenoy N, Udupa LA (2014). Assessment of learning styles of undergraduate medical students using the VARK questionnaire and the influence of sex and academic performance. Adv Physiol Educ.

[13] Bennadi D, Kashinath KR, Bharateesh JV, Kshetrimayum N (2015). Assessing learning preferences of dental students using visual, auditory, reading-writing, and kinesthetic questionnaire. J Indian Assoc Public Health Dent.

[14] AlQahtani N, AlMoammar K, Taher S, AlBarakati S, AlKofide E (2018). Learning preferences among dental students using the VARK questionnaire: a comparison between different academic levels and gender. J Pak Med Assoc.

[15] Asiry MA (2016). Learning styles of dental students. Saudi J Dent Res.

